# Identification of discriminant features from stationary pattern of nucleotide bases and their application to essential gene classification

**DOI:** 10.3389/fgene.2023.1154120

**Published:** 2023-04-20

**Authors:** Ranjeet Kumar Rout, Saiyed Umer, Monika Khandelwal, Smitarani Pati, Saurav Mallik, Bunil Kumar Balabantaray, Hong Qin

**Affiliations:** ^1^ National Institute of Technology Srinagar, Hazratbal, Jammu and Kashmir, India; ^2^ Aliah University, Kolkata, West Bengal, India; ^3^ Dr. B R Ambedkar National Institute of Technology Jalandhar, Jalandhar, Punjab, India; ^4^ Harvard T H Chan School of Public Health, Boston, United States; ^5^ Department of Pharmacology and Toxicology, University of Arizona, Tucson, AZ, United States; ^6^ National Institute of Technology Meghalaya, Shillong, Meghalaya, India; ^7^ Department of Computer Science and Engineering, University of Tennessee at Chattanooga, Chattanooga, TN, United States

**Keywords:** essential genes, DNA, co-occurrence matrix, feature analysis, classification

## Abstract

**Introduction:** Essential genes are essential for the survival of various species. These genes are a family linked to critical cellular activities for species survival. These genes are coded for proteins that regulate central metabolism, gene translation, deoxyribonucleic acid replication, and fundamental cellular structure and facilitate intracellular and extracellular transport. Essential genes preserve crucial genomics information that may hold the key to a detailed knowledge of life and evolution. Essential gene studies have long been regarded as a vital topic in computational biology due to their relevance. An essential gene is composed of adenine, guanine, cytosine, and thymine and its various combinations.

**Methods:** This paper presents a novel method of extracting information on the stationary patterns of nucleotides such as adenine, guanine, cytosine, and thymine in each gene. For this purpose, some co-occurrence matrices are derived that provide the statistical distribution of stationary patterns of nucleotides in the genes, which is helpful in establishing the relationship between the nucleotides. For extracting discriminant features from each co-occurrence matrix, energy, entropy, homogeneity, contrast, and dissimilarity features are computed, which are extracted from all co-occurrence matrices and then concatenated to form a feature vector representing each essential gene. Finally, supervised machine learning algorithms are applied for essential gene classification based on the extracted fixed-dimensional feature vectors.

**Results:** For comparison, some existing state-of-the-art feature representation techniques such as Shannon entropy (SE), Hurst exponent (HE), fractal dimension (FD), and their combinations have been utilized.

**Discussion:** An extensive experiment has been performed for classifying the essential genes of five species that show the robustness and effectiveness of the proposed methodology.

## 1 Introduction

Essential genes are necessary for the survival of a living being and are considered the basis of life. Essential genes consist of vital data of genomes and, hence, could be the key to the broad interpretation of life and expansion (Juhas et al., 2011). It decides significant attributes involving cellular structure, chemistry, and reproduction, among others. Genomes have encoded data for the functions regularly viewed as in all life forms, and the instructions could be species-specific. Some genes appear essential for survival, whereas others seem to be optional. Essential genes have been provided to segregate genes and determine the fundamental sustaining cellular life components. Deletion of an essential gene would result in cell death. As a result, essential gene prediction aids in identifying the bare minimum of genes necessary for the vital survival of specific cell types. The discovery and analysis of essential genes aids our understanding of origin of life (Koonin, 2000). Furthermore, essential genes play a crucial role in synthetic molecular biology, vital to genome development. An extensive comprehension of essential genes can empower researchers to clarify the biological essence of microorganisms (Juhas et al., 2014), generate the smallest genome subset (Itaya, 1995), evolve promising medication targets, and create probable drugs to fight infectious diseases (Dickerson et al., 2011). Due to their significance, the identification of essential genes has been viewed as essential in bioinformatics and genomics.

Essential genes are a set of genes necessary for an organism to thrive in a certain climate. Most of these are only necessary for particular circumstances. For instance, if a cell is supplied with the amino acid lysine, the gene responsible for lysine production is non-essential. However, if the amino acid supply is unavailable, the gene encoding the enzyme responsible for lysine biosynthesis becomes essential, as protein synthesis is not possible without it. Essential genes regulate the activity of fundamental cells in almost every species (Qin, 2019; Guo et al., 2021). Genes are essential if they cannot be knocked out individually under circumstances when most of the needed nutrients are present in the growth medium and the organism grows at its optimal temperature. One of the major issues is determining which identified genes are necessary. There are various experimental techniques to identify essential genes in microorganisms, such as gene knockouts (Roemer et al., 2003), RNA interference (Cullen and Arndt, 2005), transposon mutagenesis (Veeranagouda et al., 2014), and single-gene knockout procedures (Giaever et al., 2002). However, these experimental techniques have various benefits and are generally good. They are still expensive and laborious. So, there is a need for computational methods to identify essential genes.

Because essential genes have biological significance, several computational methods, particularly machine learning methods, have been employed to ascertain them. For this objective, many feature extraction and model building approaches have been developed (Gil et al., 2004; McCutcheon and Moran, 2010; Juhas et al., 2012; Mobegi et al., 2017). Chen and Xu (2005) effectively used high-throughput data and machine learning techniques in *Saccharomyces cerevisiae* to evaluate protein dispensability. Seringhaus et al. (2006) constructed a machine learning model to predict essential genes in *S. cerevisiae* using several intrinsic genomic factors. Additionally, Yuan et al. (2012) designed three machine learning techniques based on informative genomic characteristics to detect knockdown lethality in mice. Deng (2015) proposed an important gene classification algorithm using hybrid characteristics like intrinsic and context-dependent genome aspects. This model acquired area under the receiver operating characteristic curve (AUC) scores of 0.86–0.93 when testing the same organism and scores of 0.69–0.89 when predicting cross-organisms using ten-fold cross-validation.

Zhang et al. (2020) have contributed significantly by combining sequence- and network-based features to identify essential genes and arrived at valid results by utilizing a deep learning-based model to learn the characteristics generated from sequencing data and protein–protein interaction networks. Liu et al. (2017) published the findings of comprehensive research on 31 bacterial species, including cross-validation, paired, self-test, and leave-one-species-out experiments. Rout et al. (2020) proposed a method to identify essential genes of four species based on various quantitative methods, including purine and pyrimidine distribution. Le et al. (2020) proposed a model for identifying essential genes using an ensemble deep neural network. Xu et al. (2020) developed a method to predict essential genes in prokaryotes based on sequence-based features using an artificial neural network. A web server, Human Essential Genes Interactive Analysis Platform (HEGIAP), was developed by Chen et al. (2020) for detailed analysis of human essential genes.

An expression-based predictor was developed by Kuang et al. (2021) to recognize the essential genes in humans. The predictor utilized gene expression profiles to predict lncRNAs in cancer cells. Senthamizhan et al. (2021) created a database NetGenes for essential genes, which contains predictions for 2,711 bacterial species using network-based features. The protein–protein interaction network was used to extract features from the STRING database. Marques de Castro et al. (2022)predicted the essential genes in Tribolium castaneum and Drosophila melanogaster based on the physicochemical and statistical data along with subcellular locations. They extracted extrinsic and intrinsic attributes from the essential and nonessential data. This paper analyzed the DNA sequences of five species, i.e., Homo sapiens, Danio rerio, D. melanogaster, Mus musculus, and Arabidopsis thaliana, to identify essential genes. The proposed model extracts co-occurrence matrices from the essential gene sequences to find some informative patterns that distinguish the species. This paper also finds the impact of different co-occurrence matrices and existing features, such as Hurst exponent (HE), fractal dimension (FD), Shannon entropy (SE), and modified Shannon entropy (MSE).

The rest of the paper is structured in the following manner. The definitions of various fundamental parameters are given in Section 2, with relevant descriptions. The proposed methodology with detailed dataset description is discussed in Section 3. The efficiency of our strategy is proven by experimental findings and comments in Section 4, which summarizes the paper by highlighting the most important aspects of the whole investigation. Finally, the paper is concluded in Section 5.

## 2 Basic terminology

Essential genes are a family linked to critical cellular activities for survival of species. Identifying essential genes is a multidisciplinary process that necessitates both computational and wet-lab validation experiments. Several machine learning methods have been developed to improve classification accuracy, making it a time-consuming and resource-intensive process. Hence, with lower validation costs, most of these methods use supervised methods, which necessitate massive labeled training data sets, typically impractical for less-sequenced species. On the other hand, the rise of high-throughput wet-lab experimental approaches like next-generation sequencing has resulted in an oversupply of unlabeled essential gene sequence data. In the initial study, it has been observed that a fixed-dimensional feature vector represents every DNA sequence by using various quantitative measures, such as SE, MSE, FD, and HE. To estimate these quantitative measures, we convert gene sequences into binary sequences based on pyrimidine and purine distribution. The two main forms of nucleotide bases in DNA are made up of nitrogenous bases. Adenine (A) and guanine (G) are purines, whereas cytosine (C) and thymine (T) are pyrimidines. Here, purine and pyrimidine bases are expressed as 1 and 0, respectively.

A/G→1and C/T→0. (1)

### 2.1 Shannon entropy and modified Shannon entropy

SE may be used to determine how much uncertainty or information a sequence contains (Zurek, 1989; Khandelwal et al., 2022b). The uncertainty affects the distribution of each word. A sequence’s uncertainty concerning a base pair ranges from 0 to 2n, where n is the length of a word. The SE uses the probability p of the two possibilities (0/1) to calculate information entropy. The following equation gives the SE of a binary sequence:

$$\mathrm{SE}=-\overset{1}{\underset{i=0}{\sum }}p_{i}\log_{2}\left(p_{i}\right),$$ (2)

where *p*_*i*_ indicates the probability of two values regarding the binary sequence, and SE is used to compute the uncertainty in a binary string (Khandelwal et al., 2022a). When the probability *p* = 0, the event is assured never to happen, resulting in no uncertainty and entropy of 0. Similarly, if *p* = 1, the result is definite; hence, the entropy must be 0. When *p* = 1/2, the uncertainty is highest, and the SE is 1. The MSE of different word size is given by

$$MSE=-\overset{k}{\underset{j=1}{\sum }}w_{j}\log_{2}\left(w_{j}\right),$$ (3)

where *w*_*j*_ indicates the frequency of the *j* ^*th*^ word in the gene sequence. For instance, for a word of length 1, *w*_*j*_ is determined using the frequencies of purine or pyrimidine 0, 1, and for a word of length 2, *w*_*j*_ is determined using the two-time repeat of purine or pyrimidine 00, 10, 01, and 11. The number of words determined by taking the maximum length of both purines and pyrimidines is represented by k (Rout et al., 2020).

### 2.2 Hurst exponent

The HE evaluates a data set’s smoothness and degree of similarities. The HE is often used to analyze auto-correlation in time-series analysis. It is calculated using rescaled range analysis (R/S analysis) and has a value of 0–1 (Hurst, 1951; Khandelwal et al., 2022c). A negative auto-correlation of a time series is indicated by a HE value between 0 and 0.5, while a HE value between 0.5 and 1 indicates a positive auto-correlation. If the HE value is 0.5, the series is random, meaning that there is no relation between the variable and its previous values (Hassan et al., 2021; Rout et al., 2022). The HE of a binary sequence *D*_*n*_ is computed by the following equation:

$$\frac{R\left(n\right)}{S\left(n\right)}={\left(\frac{n}{2}\right)}^{HE},$$ (4)

where

$$S\left(n\right)=\sqrt{\frac{1}{n}\overset{n}{\underset{i=1}{\sum }}{\left(D_{i}-m\right)}^{2}},$$ (5)

and

$$R\left(n\right)=\max\left(X_{1},X_{2},\ldots ,X_{n}\right)-\min\left(X_{1},X_{2},\ldots ,X_{n}\right),$$ (6)

$$X_{t}=\overset{t}{\underset{i=1}{\sum }}\left(D_{i}-m\right)\;for\;\;t=1,2,3,\ldots ,n$$ (7)

$$m=\frac{1}{n}\overset{n}{\underset{i=1}{\sum }}D_{i}.$$ (8)

### 2.3 Fractal dimension

Every DNA sequence is converted into indicator matrices (Rout et al., 2018; Umer et al., 2021). Let X = {A, T, C, and G} denote the set of finite alphabet nucleotides, and D(N) denote a DNA sequence with four symbols from X of length N. The indicator function for every DNA sequence is described by the following equation:

$$F:D\left(N\right)\times D\left(N\right)\to \left\{0,1\right\},andD\left(N\right)=\left\{0,1\right\},$$ (9)

such that the indicator matrix will be

$$I\left(N,N\right)=\left\{\begin{array}{cc} 1, & \text{if }s_{i}=s_{j}\\ 0, & \text{if }s_{i}\neq s_{j} \end{array}\right.\;where\;\;s_{i},s_{j}\in D\left(N\right).$$ (10)

Here, I(N, N) is a matrix with values 0 and 1, and it produces a binary image of the DNA sequence as a 2D dot-plot. Within the same sequence, the binary image can represent the distribution of 0s and 1s. It is possible to assign a white dot to 0 and a black dot to 1. The FD from an indicator matrix can be computed as the average number of *σ*(*n*) of 1, randomly selected n× n from an N× N indicator matrix (Cattani, 2010; Rout et al., 2014; Upadhayay et al., 2019). Using *σ*(*n*), the FD is computed by the following equation:

$$FD=-\frac{1}{N}\overset{N}{\underset{n=2}{\sum }}\frac{\log\left(\sigma \left(n\right)\right)}{logn}.$$ (11)

## 3 Proposed scheme

In this paper, we used the Database of Essential Genes (http://www.essentialgene.org/) for experimental findings and discussion. This dataset consists of essential genes of five species. There are 2,051 *H. sapiens* (HS), 315 *D. rerio* (DR), 339 *D. melanogaster* (DOM), 356 *A. thaliana* (AT), and 125 *M. musculus* (MM) essential genes. Table 1 lists some of the terminologies employed in the proposed technique for reference.

**TABLE 1:** List of species considered in the proposed technique.

Name	Symbol used
*Arabidopsis thaliana*	AT
*Drosophila melanogaster*	DOM
*Danio rerio*	DR
*Homo sapiens*	HS
*Mus musculus*	MM
Naming convention for *Arabidopsis thaliana*	[*AT*_1_ − *AT*_356_]
Naming convention for *Drosophila melanogaster*	[*DOM*_1_ − *DOM*_339_]
Naming convention for *Danio rerio*	[*DR*_1_ − *DR*_315_]
Naming convention for *Homo sapiens*	[*HS*_1_ − *HS*_2051_]
Naming convention for *Mus musculus*	[*MM*_1_ − *MM*_125_]

### 3.1 Proposed feature representation technique

The DNA (deoxyribonucleic acid) sequence of essential genes S is composed of four bases: adenine (A), guanine (G), cytosine (C), and thymine (T). So, several occurrences may exist with combinations of *A*, *C*, *T*, *G* within the sequence S . The co-occurrences of *A*, *C*, *T*, *G* in the DNA sequence establishes the relationship between the nucleotide. It is the first time that a method has been proposed for finding the co-occurrences of nucleotides *A*, *C*, *T*, *G* within S . The objective of finding these co-occurrences is to analyze the patterns of *A*, *C*, *T*, *G* within the DNA sequence S to derive some useful features that uniquely discriminate the species by the feature representation of their essential genes. Assuming *x* = (*A*, *C*, *T*, *G*) is a vector of the nucleotides, then the possibility of arrangement of these characters in the DNA gene sequences is represented through co-occurrence matrices formed by the vector combination, which are shown in Table 2.

**TABLE 2:** Possible sets of occurrences of nucleobases *A*, *C*, *T*, *G* in a DNA sequence or essential gene formed by the combination of vectors, where *I*, *J*, *K*, *L*, *M*, *N*, *O*, *P* are the co-occurrence matrices.

*X*	*Y*	*X* ^*T*^ × *Y*
*X*_1_ = (*A*, *C*, *T*, *G*)	(*A*, *C*, *T*, *G*)	$\underset{4\times 4}{I}=\underset{4\times 1}{{X_{1}}^{T}}\times \underset{1\times 4}{Y}$
*X*_2_ = (*AA*, *CC*, *TT*, *GG*)	(*A*, *C*, *T*, *G*)	$\underset{4\times 4}{J}=\underset{4\times 1}{{X_{2}}^{T}}\times \underset{1\times 4}{Y}$
*X*_3_ = (*AC*, *AT*, *AG*, *CT*, *CG*, *TG*)	(*A*, *C*, *T*, *G*)	$\underset{6\times 4}{K}=\underset{6\times 1}{{X_{3}}^{T}}\times \underset{1\times 4}{Y}$
*X*_4_ = (*CA*, *TA*, *GA*, *TC*, *GC*, *GT*)	(*A*, *C*, *T*, *G*)	$\underset{6\times 4}{L}=\underset{4\times 1}{{X_{4}}^{T}}\times \underset{1\times 4}{Y}$
*X*_5_ = (*ACT*, *ACG*, *ATG*, *CTG*)	(*A*, *C*, *T*, *G*)	$\underset{4\times 4}{M}=\underset{4\times 1}{{X_{5}}^{T}}\times \underset{1\times 4}{Y}$
*X*_6_ = (*CAT*, *CAG*, *TAG*, *TCG*)	(*A*, *C*, *T*, *G*)	$\underset{4\times 4}{N}=\underset{4\times 1}{{X_{6}}^{T}}\times \underset{1\times 4}{Y}$
*X*_7_ = (*ATC*, *AGC*, *AGT*, *CGT*)	(*A*, *C*, *T*, *G*)	$\underset{4\times 4}{O}=\underset{4\times 1}{{X_{7}}^{T}}\times \underset{1\times 4}{Y}$
*X*_8_ = (*TCA*, *GCA*, *GTA*, *GTC*)	(*A*, *C*, *T*, *G*)	$\underset{4\times 4}{P}=\underset{4\times 1}{{X_{8}}^{T}}\times \underset{1\times 4}{Y}$

Here, the computed co-occurrence matrices of different combinations of nucleobases represent the distribution of nucleobases throughout the essential gene S. This distribution of nucleobases examines the texture pattern and considered the spatial relationship of nucleobases in the essential gene S. Experimentally, it has been observed that the occurrences of the spatial relationship of nucleobases cannot provide fixed information of the stationary and non-stationary patterns of A, C, T, and G. However, the obtained spatial relationship contains the information of both these patterns at a time. Hence, statistically it is easier to compute information considering both stationary and non-stationary patterns at a time rather than differentiating stationary and non-stationary patterns in S. The essential genes are very critical for the survival of any organism. It is beneficial for cell growth. Each gene sequence is variable in length, and the arrangements *A*, *C*, *T*, *G* nucleobases are zigzag. Hence, finding the stationary and non-stationary patterns of *A*, *C*, *T*, *G* and the co-occurrences of the different combinations of these nucleobases will help find its natural pattern in the gene. Hence, deriving the valuable patterns of the variety of *A*, *C*, *T*, *G* through co-occurrence matrix descriptors will considerably improve the retrieval performance and be eligible to analyze the statistical and structural information effectively from those patterns. Hence, inspired by the co-occurrence matrix of texture analysis (Umer et al., 2016) of image processing and pattern recognition, we have employed the ideas of gray-level co-occurrence matrix. Here, we have computed several co-occurrence matrices from each essential gene data. Now, $\underset{4\times 4}{I}$ , $\underset{4\times 4}{J}$ , $\underset{6\times 4}{K}$ , $\underset{6\times 4}{L}$ , $\underset{4\times 4}{M}$ , $\underset{4\times 4}{N}$ , $\underset{4\times 4}{O}$ , and $\underset{4\times 4}{P}$ co-occurrences matrices are computed that contain several patterns of *A*, *C*, *T*, *G* nucleobases in each DNA sequence S . These co-occurrence matrices are defined in Table 3, Supplementary Table S1, Supplementary Table S2, Supplementary Table S3, Supplementary Table S4, Supplementary Table S5, Supplementary Table S6, and Supplementary Table S7, respectively.

**TABLE 3:** Co-occurrence matrix *I* that contains several patterns of *A*, *C*, *T*, *G* nucleobases in DNA gene sequence S

	A	C	T	G
A	#(AA)	#(AC)	#(AT)	#(AG)
C	#(CA)	#(CC)	#(CT)	#(CG)
T	#(TA)	#(TC)	#(TT)	#(TG)
G	#(GA)	#(GC)	#(GT)	#(GG)

Here, from the given DNA sequence S , the aforementioned co-occurrence matrices are obtained. Each co-occurrence matrix G contains the number of occurrences of *A*, *C*, *T*, *G* nucleobases with a specific combinations and offset in S . Since a sequence S with *q* different combinations of *A*, *C*, *T*, *G* nucleobases will produce a co-occurrence matrix of size *q* × 4 for the given offset, so the (*r*,*s*)^*th*^ value of a co-occurrence matrix (Table 3, Supplementary Table S1, Supplementary Table S2, Supplementary Table S3, Supplementary Table S4, Supplementary Table S5, Supplementary Table S6, and Supplementary Table S7) gives the number of times that *r* ^*th*^ and *s* ^*th*^ nucleobases present in S . Hence, mathematically, here each co-occurrence matrix (Table 3, Supplementary Table S1, Supplementary Table S2, Supplementary Table S3, Supplementary Table S4, Supplementary Table S5, Supplementary Table S6, and Supplementary Table S7) is given by

$$G=\overset{n}{\underset{i=1}{\sum }}\overset{n}{\underset{j=1}{\sum }}\left\{\begin{array}{cc} 1\; & G_{\left(i,j\right)}=r\;\&\;G_{\left(i+△i,j+△j\right)}=s\\ 0\; & \text{otherwise} \end{array},\right.$$ (12)

The offset (△*i*, △*j*) defines the spatial relation for which the matrix G is calculated. The number of co-occurrences of the combinations of *A*, *C*, *T*, *G* present in S is obtained by the co-occurrence matrices. So, to extract distinguish and discriminant features, each matrix G is normalized to $G^{\prime }=\frac{G}{\sum_{r=0}^{q}\sum_{s=0}^{q}G\left(r,s\right)}$ . Then, the normalized co-occurrence matrix $G^{\prime }$ is used to compute some features like entropy, dissimilarity, energy, homogeneity, and contrast. The mathematical definitions of these features are shown in Table 4.

**TABLE 4:** Features extracted from a co-occurrence matrix G of DNA sequence *S*.

Feature	Formulae
Energy	$\sum_{r=0}^{q}\sum_{s=0}^{q}G^{\prime }{\left(r,s\right)}^{2}$
Entropy	$\sum_{r=0}^{q}\sum_{s=0}^{q}-G^{\prime }\left(r,s\right)\times \ln\left(G^{\prime }\left(r,s\right)\right)$
Homogeneity	$\sum_{r=0}^{q}\sum_{s=0}^{q}\frac{G^{\prime }\left(r,s\right)}{\left(1+{\left(r-s\right)}^{2}\right)}$
Contrast	$\sum_{r=0}^{q}\sum_{s=0}^{q}G^{\prime }\left(r,s\right)\times {\left(r-s\right)}^{2}$
Dissimilarity	$\sum_{r=0}^{q}\sum_{s=0}^{q}G^{\prime }\left(r,s\right)\times |\left(r-s\right)|$

Now, the features defined in Table 4 are extracted from each co-occurrence matrix (Table 3, Supplementary Table S1, Supplementary Table S2, Supplementary Table S3, Supplementary Table S4, Supplementary Table S5, Supplementary Table S6, and Supplementary Table S7), and the list of feature vectors extracted from these matrices is obtained as follows:

*f*_*I*_ = (*f*_1_, *f*_2_, *f*_3_, *f*_4_, *f*_5_) from *I* (Table 3)

*f*_*J*_ = (*f*_6_, *f*_7_, *f*_8_, *f*_9_, *f*_10_) from *J* (Supplementary Table S1)

*f*_*K*_ = (*f*_11_, *f*_12_, *f*_13_, *f*_14_, *f*_15_) from *K* (Supplementary Table S2)

*f*_*L*_ = (*f*_16_, *f*_17_, *f*_18_, *f*_19_, *f*_20_) from *L* (Supplementary Table S3)

*f*_*M*_ = (*f*_21_, *f*_22_, *f*_23_, *f*_24_, *f*_25_) from *M* (Supplementary Table S4)

*f*_*N*_ = (*f*_26_, *f*_27_, *f*_28_, *f*_29_, *f*_30_) from *N* (Supplementary Table S5)

*f*_*O*_ = (*f*_31_, *f*_32_, *f*_33_, *f*_34_, *f*_35_) from *O* (Supplementary Table S6)

*f*_*P*_ = (*f*_36_, *f*_37_, *f*_38_, *f*_39_, *f*_40_) from *P* (Supplementary Table S7)

Hence, the final feature representation of a DNA sequence or essential gene S is given by the feature vector *f* = (*f*_*I*_, *f*_*J*_, *f*_*K*_, *f*_*L*_, *f*_*M*_, *f*_*N*_, *f*_*O*_, *f*_*P*_).

### 3.2 Classification

In this study, for the classification of the essential genes in the employed species, the decision tree (DT), k-nearest neighbor (KNN), and support vector machine (SVM) classifiers are used. During experimentation, the datasets of each species *Arabidopsis thaliana* (AT), *Drosophila melanogaster* (DOM), *Danio rerio* (DR), *Homo sapiens* (HS), and *Mus musculus* (MM) are divided into two, with 50% of its data input into the training set and the remaining 50% into the testing set. Then, a five-fold cross-validation technique is employed. Finally, the average performance for the testing data is reported for the proposed system.

DT is a supervised algorithm, and it is generated by using the Iterative Dichotomiser 3 algorithm (ID3) or CART algorithm (Classification algorithm and Regression Tree) (Quinlan, 1986). The DT uses decision nodes to split the dataset into smaller subsets based on information gain (IG) or the Gini index. ID3 uses IG to evaluate how well an attribute splits the training dataset based on its classification objective. IG is the difference between the dataset’s entropy before and after splitting depending on the specified attribute values. Let X = *x*_1_, *x*_2_, *x*_3_, …., *x*_*n*_ represent the set of instances, A represent the attribute, and *X*_*v*_ subset of X having A = v. Then, IG is given by

$$IG\left(X,A\right)=Ent\left(X\right)-\underset{v\in V\left(A\right)}{\sum }\frac{\left|X_{v}\right|}{\left|X\right|}\cdot Ent\left(X_{v}\right),$$ (13)

where ENT(X) is the entropy of X and V(A) is the collection of all possible A values. Entropy of X is given by

$$Ent\left(X\right)=\overset{c}{\underset{i=1}{\sum }}-p_{i}\log_{2}p_{i},$$ (14)

where *p*_*i*_ denotes the probability for current state X.

KNN is a supervised machine learning and non-parametric technique that signifies that it makes no assumptions about the underlying data. The KNN method ensures that the unseen data and existing dataset are comparable and places the unseen data in the most similar class to the unseen data. KNN works by just storing the data during training time. When it sees new data at testing time, it finds k-nearest neighbor to the latest data by using distance measure, i.e., Euclidean distance, and classifies it based on the similarity (Peterson, 2009). The steps of the KNN algorithm are as follows.

1. First, select the value of K, i.e., the closest data points. Any integer may be used as K.
2. Do the following for each data point in the test data set: (i) find the distance between the data point and all samples in the training dataset using one of the following methods: Manhattan, Euclidean, or Hamming distance. In this paper, Euclidean distance measure is used for calculating the distance; (ii) sort samples in the ascending order depending on the distance value; (iii) select the top K samples as the nearest neighbors to the test data point; (iv) next, the test data point will be assigned a class depending on the most common class of these K samples.

The SVM is a supervised machine learning approach for classifying data. The SVM is a well-known technique used in various bioinformatics and computational biology problems, and it needs fewer model parameters to describe the non-linear transition from primary sequence to protein structure region. To minimize the error, the SVM will create the hyperplane repeatedly. The SVM is noted for its quick training, which is necessary for high-throughput database testing (Suthaharan, 2016). Let the dataset be represented by (*X*_1_, *y*_1_), (*X*_2_, *y*_2_), (*X*_3_, *y*_3_), …. , (*X*_*n*_, *y*_*n*_). The SVM solves the following equation:

$$\underset{w,b}{\min}\|w{\|}^{2}\text{such that}\forall i,y_{i}\left(\left\langle w,X_{i}\right\rangle +b\right)\geq 1,$$ (15)

where w and b is the weight and bias of the hyperplane equation *w* ⋅ *X* + *b* = 0, respectively.

### 3.3 Evaluation metrics

In this paper, the essential gene classification problem is a multi-class classification problem as we have classified essential genes of five species, i.e., AT, DOM, DR, HS, and MM. For every class in the target, the evaluation matrices (accuracy, precision, recall, and F1-score) were computed. Then, the weighted averaging technique was used to give the final value of evaluation metrics.

$$Accuracy=\frac{\sum_{i=1}^{C}n_{i}\times \frac{TP_{i}+TN_{i}}{TP_{i}+TN_{i}+FP_{i}+FN_{i}}}{\sum_{i=1}^{C}n_{i}},$$ (16)

$$Precision=\frac{\sum_{i=1}^{C}n_{i}\times \frac{TP_{i}}{TP_{i}+FP_{i}}}{\sum_{i=1}^{C}n_{i}}$$ (17)

$$Recall=\frac{\sum_{i=1}^{C}n_{i}\times \frac{TP_{i}}{TP_{i}+FN_{i}}}{\sum_{i=1}^{C}n_{i}}$$ (18)

$$F1-score=\frac{\sum_{i=1}^{C}n_{i}\times \frac{2\times Precision_{i}\times Recall_{i}}{Precision_{i}+Recall_{i}}}{\sum_{i=1}^{C}n_{i}},$$ (19)

where

$$Precision_{i}=\frac{TP_{i}}{TP_{i}+FP_{i}},$$ (20)

and

$$Recall_{i}=\frac{TP_{i}}{TP_{i}+FN_{i}},$$ (21)

where *TP*_*i*_, *TN*_*i*_, *FP*_*i*_, and *FN*_*i*_ are the counts of true positives, true negatives, false positives, and false negatives, respectively, for the *i* ^*th*^ class. Here, *C* represents the number of classes in the problem, and *n*_*i*_ indicates the number of samples in the *i* ^*th*^ class.

### 3.4 Model framework

**FIGURE 1:**
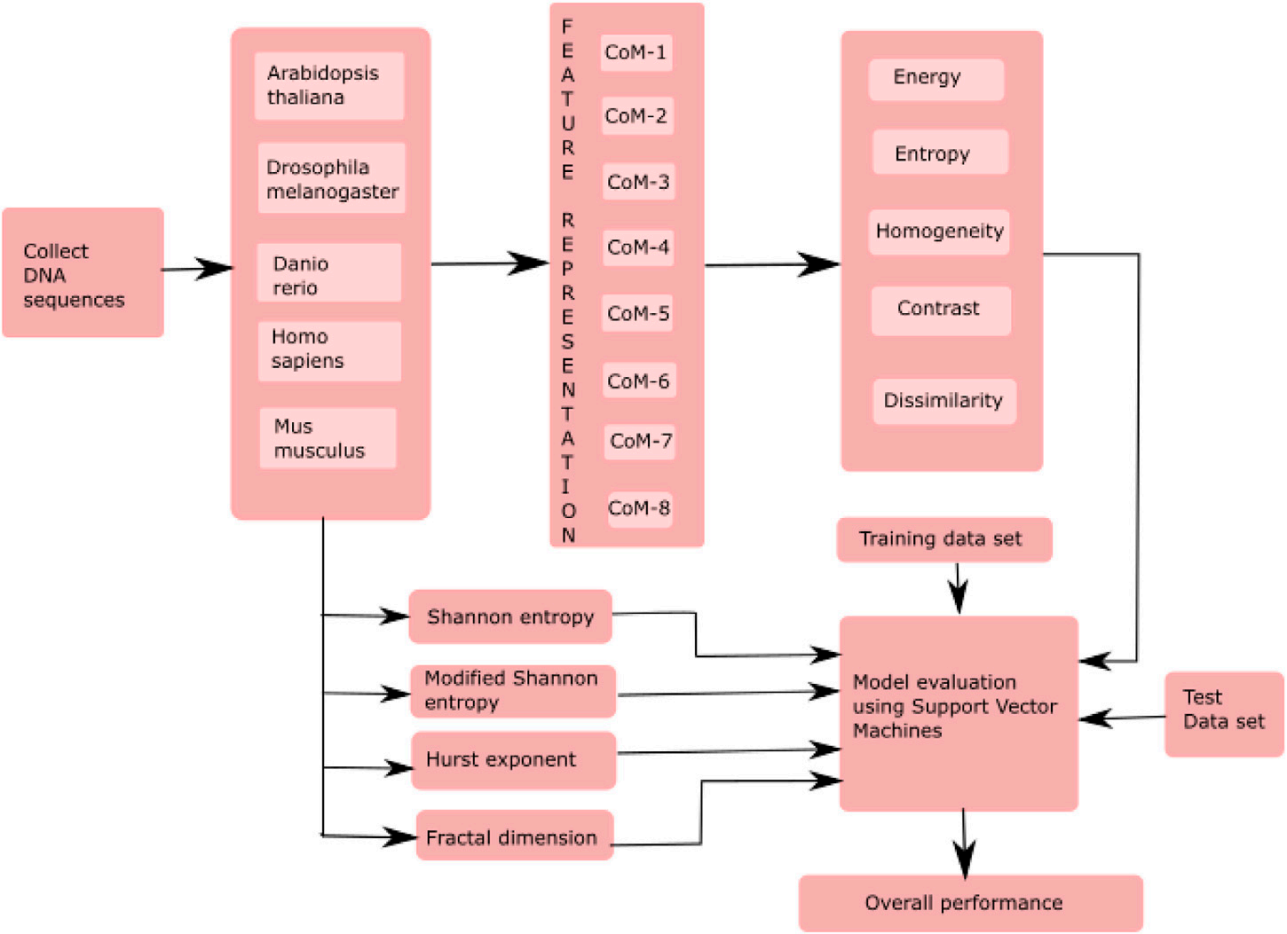
Framework of the proposed model for the classification of essential genes. Here, CoM indicates the co-occurrence matrices.

The proposed model classified essential genes of five species based on co-occurrence matrices. The proposed model finds the eight different co-occurrence matrices from the DNA sequences. From each co-occurrence matrix, five features, i.e., energy, entropy, homogeneity, contrast, and dissimilarity, were extracted. The existing features, such as HE, FD, SE, and MSE were also computed and then combined with the proposed features for the classification of essential genes. A supervised machine learning algorithm, SVM, was used to evaluate the model. Figure 1 shows essential genes. A supervised machine learning algorithm, SVM was used to evaluate the model. Figure 1 shows the framework of the proposed model.

## 4 Result and discussion

The proposed essential gene classification model can identify novel essential genes with high recall and precision while only requiring a small number of previously identified essential genes in some species. Such a method could be highly beneficial when investigating essential genes in newly sequenced genomes of other species with few known examples of essential genes. The proposed work has been implemented in the ‘Python’ environment, while the ‘Python’ library of machine learning algorithms has been employed for data classification tasks. Python is the best scripting and programming language, is open-source, and has high-level object-oriented programming approaches that deal with mathematical and statistical functions. The method’s implementation for the proposed methodology is executed in the Kaggle repository that explores research to data scientists and machine learning engineers as best practitioners in these fields. Here, for Python tools, we have employed NumPy, Pandas, Matplotlib, Sklearn.Preprocessing, Sklearn.Classifiers, Sklearn.Metrics, and some other packages for data analysis and prediction models. The feature vectors extracted from each DNA gene sequence S undergo KNN, DT, and SVM classifiers. The datasets from AT, DOM, DR, HS, and MM species are given in Table 5. The experimentation of the proposed methodology has been divided into sub-sections.

**TABLE 5:** Demonstration of actual files containing gene sequences corresponding to AT, DOM, DR, HS, and MM species.

	Actual files	Actual files containing DNA sequences
AT	356	356
DOM	339	339
DR	315	315
HS	2054	2051
MM	411	125

### 4.1 Experiment for the proposed features

**FIGURE 2:**
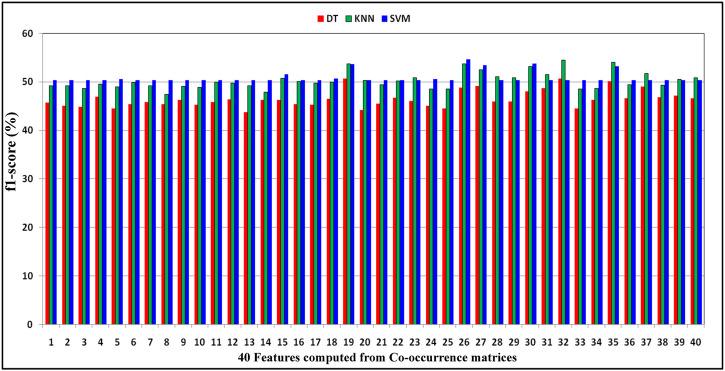
Demonstration of distribution of F1-score performance obtained by decision tree, KNN, and SVM classifiers with respect to the 40 features computed from co-occurrence matrices of DNA gene sequence *S*.

In this section, experiments with individual features have been performed. Here, from each DNA sequence S , individual feature from each *f*_*I*_, *f*_*J*_, *f*_*K*_, *f*_*L*_, *f*_*M*_, *f*_*N*_, *f*_*O*_, *f*_*P*_ have been considered, and then classification has been performed. Figure 2 demonstrates the distribution of F1-score performance obtained by DT, KNN, and SVM classifiers with respect to every 40 features computed from co-occurrence matrices of DNA sequence *S*. From this figure, it has been observed that both the KNN and SVM classifiers predict the classification problem better than the DT classifier for most of the features. Moreover, it has also been observed that classifiers have obtained more or less similar performance for most features but better performance due to the 19th, 26th, 27th, 30th, 32nd, and 35th features of the forty-dimensional feature vector *f*. For measuring the impact of individual features such as entropy, homogeneity, energy, contrast, and dissimilarity on the classification of essential genes, the performance has been reported concerning KNN, DT, and SVM classifiers in Table 6. Here, experiments are carried out under the same training–testing protocols, and from each DNA sequence S , the corresponding features are extracted from all co-occurrence matrices. So, each eight-dimensional feature vector is extracted for entropy, homogeneity, energy, contrast, and dissimilarity features.

**TABLE 6:** Impact of different co-occurrence features on the classification of essential gene sequences of AT, DOM, DR, HS, and MM species.

Classifier	Accuracy	Precision	Recall	F1-score
Effect of entropy features				
K-nearest neighbors	63.56	56.68	63.56	59.39
Decision tree	52.95	53.56	52.95	53.25
Support vector machine	64.37	41.44	64.37	50.42
Effect of dissimilarity features				
K-nearest neighbors	62.96	57.38	62.96	59.55
Decision tree	52.70	53.84	52.70	53.25
Support vector machine	67.07	58.80	67.07	56.75
Effect of energy features				
K-nearest neighbors	59.48	52.71	59.48	55.46
Decision tree	48.65	49.82	48.65	49.22
Support vector machine	64.94	50.32	64.94	51.83
Effect of homogeneity features				
K-nearest neighbors	63.06	57.59	63.06	59.99
Decision tree	53.61	54.81	53.61	54.19
Support vector machine	67.67	60.76	67.67	58.29
Effect of contrast features				
K-nearest neighbors	64.25	58.92	64.25	61.02
Decision tree	54.80	56.27	54.80	55.51
Support vector machine	68.36	59.82	68.36	58.85

As shown in Table 6, for every feature, the performance is more or less the same, but for the KNN classifier, the performance is better than that of DT and SVM. Here, F1-score has been considered classification performance as the employed species AT, DOM, DR, HS, and MM have class imbalance problems. Furthermore, the effect of features computed from each co-occurrence matrix in the subsequent experiments has been considered. Here, the 5-dimensional feature vector is extracted from each co-occurrence matrix. The performance due to these feature vectors is reported in Table 7 under the same training–testing protocol. Table 7 shows that there is a more or less a similar effect of co-occurrence matrix features on the essential gene classification. Hence, the features computed from the co-occurrence metrics are helpful and effective. Here, the KNN classifier has better performance.

**TABLE 7:** Impact of features extracted from different co-occurrence matrices for the classification of essential gene sequences of AT, DOM, DR, HS, and MM species.

Classifier	Accuracy	Precision	Recall	F1-score
Effect of first matrix				
K-nearest neighbors	63.37	56.39	63.37	59.20
Decision tree	53.70	54.02	53.70	53.85
Support vector machine	64.38	41.44	64.38	50.42
Effect of second matrix				
K-nearest neighbors	62.05	54.43	62.05	57.54
Decision tree	53.20	53.88	53.20	53.53
Support vector machine	64.38	41.44	64.38	50.42
Effect of third matrix				
K-nearest neighbors	60.58	52.69	60.58	55.66
Decision tree	49.72	51.01	49.72	50.34
Support vector machine	64.38	41.44	64.38	50.42
Effect of fourth matrix				
K-nearest neighbors	62.96	58.32	62.96	59.41
Decision tree	54.33	55.14	54.33	54.72
Support vector machine	64.38	41.44	64.38	50.42
Effect of fifth matrix				
K-nearest neighbors	57.91	49.72	57.91	53.02
Decision tree	47.24	48.14	47.24	47.69
Support vector machine	64.38	41.44	64.38	50.42
Effect of sixth matrix				
K-nearest neighbors	61.49	54.13	61.49	57.14
Decision tree	52.69	54.34	52.69	53.49
Support vector machine	65.35	47.61	65.35	53.36
Effect of seventh matrix				
K-nearest neighbors	58.82	52.94	58.82	55.37
Decision tree	50.44	51.56	50.44	50.99
Support vector machine	64.81	46.81	64.81	53.45
Effect of eighth matrix				
K-nearest neighbors	56.12	50.86	56.12	52.78
Decision tree	49.28	49.86	49.28	49.56
Support vector machine	64.38	41.44	64.38	50.42

### 4.2 Experiment for the existing features

**FIGURE 3:**
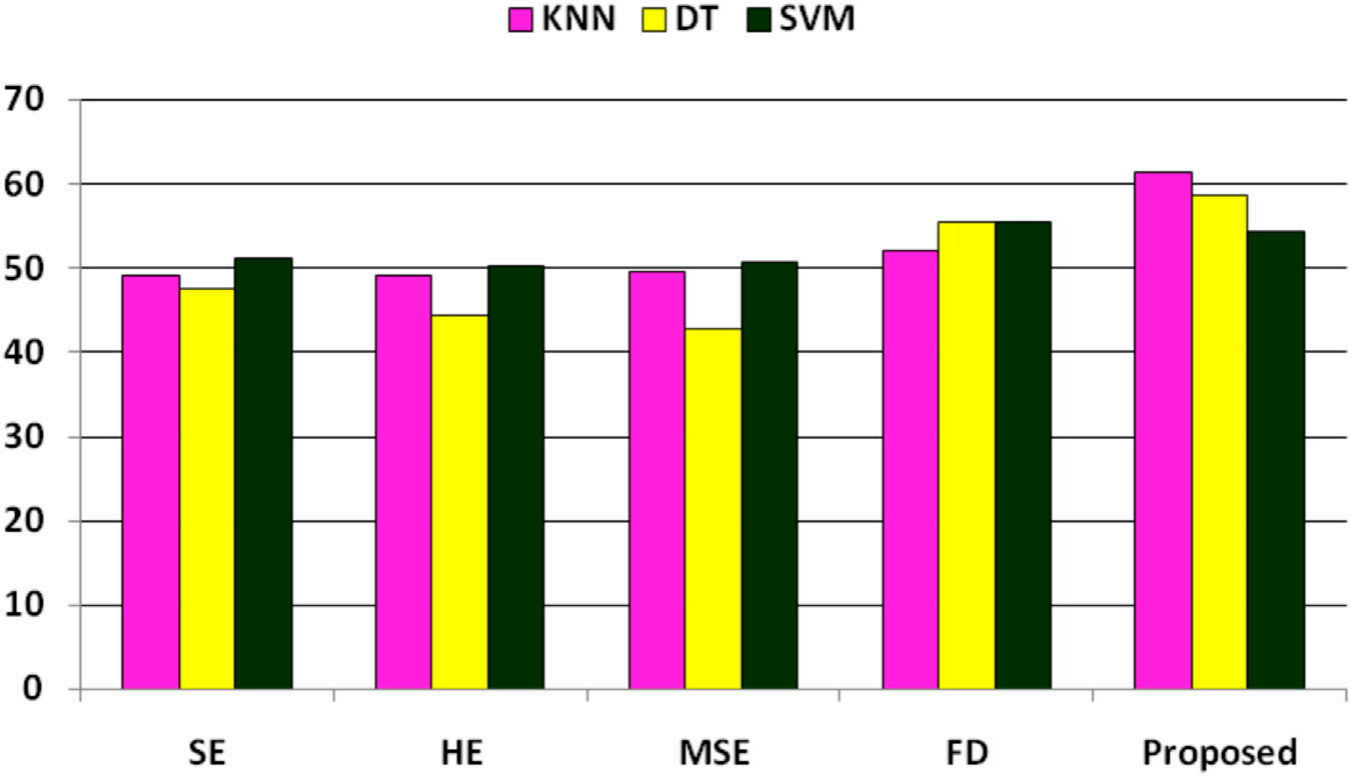
Performance (F1-score) comparison of existing features and the proposed features for the classification of essential genes of AT, DOM, DR, HS, and MM species.

In the further experiment, the performance has been compared with some existing state-of-the-art feature extraction techniques such as SE, MSE, HE, and FD(discussed in Section 2), where these features are extracted accordingly. The performance is obtained concerning KNN, DT, and SVM classifiers. The performance due to these features is reported in Table 8, implying that SE, HE, MSE, and FD features have more or less similar performance. Still, among the classifiers, SVM has obtained better performance. The comparison of these performances and the proposed system has been shown in Figure 3, which shows that the proposed approach has better classified the essential genes of AT, DOM, DR, HS, and MM species under the same training–testing protocol. Here, the difference is in the proposed system, and the forty-dimensional feature vector is considered, while the one-dimensional feature vector is extracted in each existing feature extraction technique. Hence, this work investigates the discriminatory power of co-occurrence matrix features with better performance than the existing state-of-the-art features.

**TABLE 8:** Impact of existing and proposed features on the classification of essential genes for the AT, DOM, DR, HS, and MM species.

Classifier	Accuracy	Precision	Recall	F1-score
Effect of Shannon entropy features				
K-nearest \neighbors	53.10	46.24	53.10	49.14
Decision tree	48.28	46.96	48.28	47.53
Support vector machine	64.33	41.38	64.33	50.36
Effect of Hurst exponent features				
K-nearest neighbors	53.98	45.63	53.98	49.14
Decision tree	43.57	45.41	43.57	44.45
Support vector machine	64.33	41.38	64.33	50.36
Effect of modified Shannon entropy features				
K-nearest neighbors	54.67	46.20	54.67	49.71
Decision tree	41.76	43.98	41.76	42.80
Support vector machine	64.26	45.64	64.26	50.66
Effect of fractal dimension features				
K-nearest neighbors	58.11	52.19	58.11	52.15
Decision tree	68.35	46.72	68.35	55.51
Support vector machine	68.35	46.72	68.35	55.51
Effect of proposed features				
K-nearest neighbors	64.95	59.49	64.95	61.50
Decision tree	58.31	59.24	58.31	58.70
Support vector machine	66.14	56.57	66.14	54.35

### 4.3 Experiment for the combined features

The co-occurrence of nucleotides *A*, *C*, *T*, *G* in the essential gene derives the distribution of these nucleotides and also their relative position information within the gene S . The existing state-of-the-art techniques of feature extraction (discussed in this work) are key measures in information theory. For example, SE and its modified technique compute the amount of uncertainty and randomness of nucleotides in the gene S . HE measures the relative tendency and characteristic parameters for analyzing its distribution in the essential gene. The FD computes the fractal-like distribution of nucleotides from the indicator matrix calculated from the essential gene S . So, the similarity of patterns of nucleotides computed by the co-occurrence matrices and the information of uncertainty, randomness, relative tendency, and fractal-like distribution information in S are combined here to obtain more discriminant features for the classification of essential genes of AT, DOM, DR, HS, and MM species. The principal component analysis of dimensionality reduction with variation ratio has been adopted to find the best suitable combination of these features. The performance due to the combination of these features is demonstrated in Table 9.

**TABLE 9:** Demonstration of discriminant features among proposed features, Shannon entropy, Hurst exponent, modified Shannon entropy and fractal dimension features.

Feature	Eigen-values	Rank	Feature	Eigen-values	Rank
*f*_1_	13.908	1	*f*_23_	0.283	23
*f*_2_	4.434	2	*f*_24_	0.257	24
*f*_3_	3.628	3	*f*_25_	0.224	25
*f*_4_	2.895	4	*f*_26_	0.192	26
*f*_5_	2.505	5	*f*_27_	0.152	27
*f*_6_	2.233	6	*f*_28_	0.109	28
*f*_7_	1.904	7	*f*_29_	0.041	29
*f*_8_	1.602	8	*f*_30_	0.032	30
*f*_9_	1.388	9	*f*_31_	0.027	32
*f*_10_	1.133	10	*f*_32_	0.027	31
*f*_11_	0.986	11	*f*_33_	0.023	33
*f*_12_	0.855	12	*f*_34_	0.019	34
*f*_13_	0.820	13	*f*_35_	0.015	35
*f*_14_	0.750	14	*f*_36_	0.008	36
*f*_15_	0.714	15	*f*_37_	0.006	37
*f*_16_	0.525	16	*f*_38_	0.001	43
*f*_17_	0.471	17	*f*_39_	0.001	44
*f*_18_	0.440	18	*f*_40_	0.002	42
*f*_19_	0.432	19	*f*_41_	0.003	41
*f*_20_	0.333	20	*f*_42_	0.003	40
*f*_21_	0.329	21	*f*_43_	0.004	39
*f*_22_	0.299	22	*f*_44_	0.004	38

Table 10 reports the discriminatory power of combined features with respect to various dimensional reduced features concerning KNN, DT, and SVM classifiers and shows that highest F1-score is 71.42 and it is due to the SVM classifier. As this is class imbalance problem, so F1-score performance has been reported.

**TABLE 10:** Demonstration of performance due to combination of features for the classification of essential genes of AT, DOM, DR, HS, and MM species.

Variation	Classifier	Accuracy	Precision	Recall	F1-score	Feature dimension
0.85	K-nearest neighbors	72.01	66.37	72.01	68.67	4
	Decision tree	63.09	63.63	63.09	63.34	
	Support vector machine	74.30	68.77	74.30	67.69	
0.9	K-nearest neighbors	71.52	66.77	71.52	68.94	5
	Decision tree	62.67	63.81	62.67	63.18	
	Support vector machine	75.91	69.57	75.91	70.31	
0.95	K-nearest neighbors	73.82	68.83	73.82	70.80	7
	Decision tree	63.93	64.67	63.93	64.29	
	Support vector machine	76.46	72.63	76.46	71.06	
0.99	K-nearest neighbors	73.96	68.29	73.96	70.66	9
	Decision tree	64.48	65.35	64.48	64.88	
	Support vector machine	76.32	70.56	76.32	**71.42**	

The bold value indicates the highest F1-score.

**FIGURE 4:**
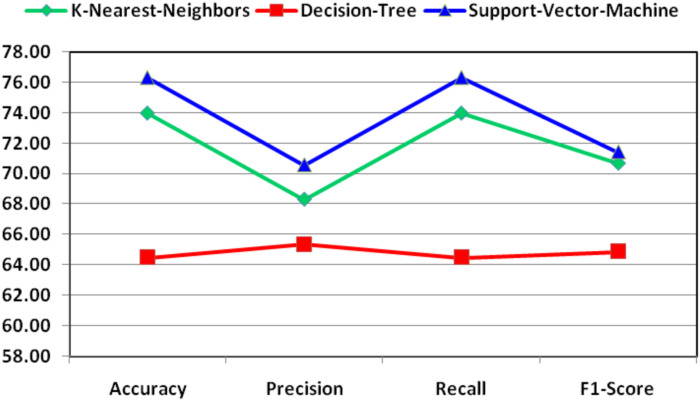
Demonstration of final performance for the combination of features for the classification of essential genes of AT, DOM, DR, HS, and MM species.

For better understanding and visibility, the final performance for the combination of features for the classification of essential genes of AT, DOM, DR, HS, and MM species has been shown in Figure 4.

## 5 Conclusion

A novel method of feature extraction and analysis for the classification of essential genes of *Arabidopsis thaliana* (AT), *Drosophila melanogaster* (DOM), *Danio rerio* (DR), *Homo sapiens* (HS), and *Mus musculus* (MM) species has been considered in this work. The implementation of the proposed scheme is divided into three segments. In the first segment, novel co-occurrence matrix-based features are extracted from genes that derive the distribution of nucleotides and their relative position from the respective gene. The features from these measures belong to the statistical analysis of the distribution of stationary patterns of nucleotides in the essential genes. In the second segment, some existing state-of-the-art feature computation techniques such as SE, HE, and FD are used as information theory measures that compute uncertainty, randomness, relative tendency, and fractal-like structures in the gene. In the third segment of this work, the features from the proposed methodology and the existing techniques are individually carried out for classification tasks where their F1-score performance has been considered for comparison. These comparisons show the robustness and effectiveness of the proposed methodology. Finally, the features from the proposed scheme and the existing techniques are combined to compute more discriminatory features for classifying essential genes of AT, DOM, DR, HS, and MM species.

## Data Availability

Data used for this study is publicly available at http://www.essentialgene.org/.
