# Supplementary material for: Identification of discriminant features from stationary pattern of nucleotide bases and their application to essential gene classification
Source: Front Genet. 2023 Apr 20;14:1154120. doi: 10.3389/fgene.2023.1154120 (PMC10156977; doi:10.3389/fgene.2023.1154120)
Supplement: Supplementary file 1 [file Table1.docx]

**Supplementary Material for Identification of Discriminant Features from Stationary Pattern of Nucleotide Bases and their Application to Essential Gene Classification**

**Supplementary Table 1: Co-occurrence matrix J that contain several patterns of A, C, T, G nucleobases in DNA gene sequence S**

|  | **A** | **C** | **T** | **G** |
| --- | --- | --- | --- | --- |
| **AA** | #(AAA) | #(AAC) | #(AAT) | #(AAG) |
| **CC** | #(CCA) | #(CCC) | #(CCT) | #(CCG) |
| **TT** | #(TTA) | #(TTC) | #(TTT) | #(TTG) |
| **GG** | #(GGA) | #(GGC) | #(GGT) | #(GGG) |

**Supplementary Table 2: Co-occurrence matrix K that contain several patterns of A, C, T, G nucleobases in DNA gene sequence S**

|  | **A** | **C** | **T** | **G** |
| --- | --- | --- | --- | --- |
| **AC** | #(ACA) | #(ACC) | #(ACT) | #(ACG) |
| **AT** | #(ATA) | #(ATC) | #(ATT) | #(ATG) |
| **AG** | #(AGA) | #(AGC) | #(AGT) | #(AGG) |
| **CT** | #(CTA) | #(CTC) | #(CTT) | #(CTG) |
| **CG** | #(CGA) | #(CGC) | #(CGT) | #(CGG) |
| **TG** | #(TGA) | #(TGC) | #(TGT) | #(TGG) |

**Supplementary Table 3: Co-occurrence matrix L that contain several patterns of A, C, T, G nucleobases in DNA gene sequence S**

|  | **A** | **C** | **T** | **G** |
| --- | --- | --- | --- | --- |
| **CA** | #(CAA) | #(CAC) | #(CAT) | #(CAG) |
| **TA** | #(TAA) | #(TAC) | #(TAT) | #(TAG) |
| **GA** | #(GAA) | #(GAC) | #(GAT) | #(GAG) |
| **TC** | #(TCA) | #(TCC) | #(TCT) | #(TCG) |
| **GC** | #(GCA) | #(GCC) | #(GCT) | #(GCG) |
| **GT** | #(GTA) | #(GTC) | #(GTT) | #(GTG) |

**Supplementary Table 4: Co-occurrence matrix M that contain several patterns of A, C, T, G nucleobases in DNA gene sequence S**

|  | **A** | **C** | **T** | **G** |
| --- | --- | --- | --- | --- |
| **ACT** | #(ACTA) | #(ACTC) | #(ACTT) | #(ACTG) |
| **ACG** | #(ACGA) | #(ACGC) | #(ACGT) | #(ACGG) |
| **ATG** | #(ATGA) | #(ATGC) | #(ATGT) | #(ATGG) |
| **CTG** | #(CTGA) | #(CTGC) | #(CTGT) | #(CTGG) |

**Supplementary Table 5: Co-occurrence matrix N that contain several patterns of A, C, T, G nucleobases in DNA gene sequence S**

|  | **A** | **C** | **T** | **G** |
| --- | --- | --- | --- | --- |
| **CAT** | #(CATA) | #(CATC) | #(CATT) | #(CATG) |
| **CAG** | #(CAGA) | #(CAGC) | #(CAGT) | #(CAGG) |
| **TAG** | #(TAGA) | #(TAGC) | #(TAGT) | #(TAGG) |
| **TCG** | #(TCGA) | #(TCGC) | #(TCGT) | #(TCGG) |

**Supplementary Table 6: Co-occurrence matrix O that contain several patterns of A, C, T, G nucleobases in DNA gene sequence S**

|  | **A** | **C** | **T** | **G** |
| --- | --- | --- | --- | --- |
| **ATC** | #(ATCA) | #(ATCC) | #(ATCT) | #(ATCG) |
| **AGC** | #(AGCA) | #(AGCC) | #(AGCT) | #(AGCG) |
| **AGT** | #(AGTA) | #(AGTC) | #(AGTT) | #(AGTG) |
| **CGT** | #(CGTA) | #(CGTC) | #(CGTT) | #(CGTG) |

**Supplementary Table 7: Co-occurrence matrix P that contain several patterns of A, C, T, G nucleobases in DNA gene sequence S**

|  | **A** | **C** | **T** | **G** |
| --- | --- | --- | --- | --- |
| **TCA** | #(TCAA) | #(TCAC) | #(TCAT) | #(TCAG) |
| **GCA** | #(GCAA) | #(GCAC) | #(GCAT) | #(GCAG) |
| **GTA** | #(GTAA) | #(GTAC) | #(GTAT) | #(GTAG) |
| **GTC** | #(GTCA) | #(GTCC) | #(GTCT) | #(GTCG) |
